# Improving data sharing in research with context-free encoded missing data

**DOI:** 10.1371/journal.pone.0182362

**Published:** 2017-09-12

**Authors:** Marieke P. Hoevenaar-Blom, Juliette Guillemont, Tiia Ngandu, Cathrien R. L. Beishuizen, Nicola Coley, Eric P. Moll van Charante, Sandrine Andrieu, Miia Kivipelto, Hilkka Soininen, Carol Brayne, Yannick Meiller, Edo Richard

**Affiliations:** 1 Department of Neurology, Academic Medical Center, University of Amsterdam, Amsterdam, The Netherlands; 2 INSERM, University of Toulouse, Toulouse, France; 3 Chronic Disease Prevention Unit, National Institute for Health and Welfare, Helsinki, Finland; 4 Department of Epidemiology and Public Health, Toulouse University Hospital, Toulouse, France; 5 INSERM, University of Toulouse UMR1027, Toulouse, France; 6 Department of General Practice, Academic Medical Centre, University of Amsterdam, Amsterdam, The Netherlands; 7 Institute of Clinical Medicine/Neurology, University of Eastern Finland, Kuopio, Finland; 8 Aging Research Center, Karolinska Institutet/ Stockholm University, Stockholm, Sweden; 9 Karolinska Institutet Center for Alzheimer Research, Stockholm, Sweden; 10 Neurocenter, neurology, Kuopio University Hospital, Kuopio, Finland; 11 Department of Public Health and Primary Care, Cambridge Institute of Public Health, University of Cambridge, United Kingdom; 12 Department of Information and Operations Management, ESCP Europe, Paris, France; 13 Department of Neurology, Radboud University Medical Center, Nijmegen, The Netherlands; Copenhagen University Hospital, DENMARK

## Abstract

Lack of attention to missing data in research may result in biased results, loss of power and reduced generalizability. Registering reasons for missing values at the time of data collection, or—in the case of sharing existing data—before making data available to other teams, can save time and efforts, improve scientific value and help to prevent erroneous assumptions and biased results. To ensure that encoding of missing data is sufficient to understand the reason why data are missing, it should ideally be context-free. Therefore, 11 context-free codes of missing data were carefully designed based on three completed randomized controlled clinical trials and tested in a new randomized controlled clinical trial by an international team consisting of clinical researchers and epidemiologists with extended experience in designing and conducting trials and an Information System expert. These codes can be divided into missing due to participant and/or participation characteristics (n = 6), missing by design (n = 4), and due to a procedural error (n = 1). Broad implementation of context-free missing data encoding may enhance the possibilities of data sharing and pooling, thus allowing more powerful analyses using existing data.

## Introduction

Missing data are often unavoidable in research, despite all efforts to reduce their occurrence in study design and conduct. Lack of attention to this important area may result in biased results, loss of power and reduced generalizability. This can seriously compromise inferences from clinical trials and observational studies.[1]

Knowing why data are missing is important to determine the most appropriate way to handle them in the analyses. The encoding of missing data should ideally be context-free—i.e. the code itself is sufficient to understand the reason why data are missing. This makes it easier to determine whether data are missing completely at random (MCAR), missing at random (MAR) or missing not at random (MNAR).[2] The information gained is particularly useful when assessing the need for various types of sensitivity analyses (if any) and when separating clearly plausible MCAR data from the rest of missing data. In the latter case this may produce more simple missing data patterns that need to be subjected to multiple imputation or alternative and equally valid methods. This again may imply that more simple methods could be used. Examples of such a situation are that family history of CVD could not be answered because of broken contact with family or that a box of questionnaires got lost. However, in the worst-case scenario, if the number of missing data is large and information on the reason why data is missing is lacking, collected data may lose their scientific value, leading to ‘research waste’.[3] Registering this information at the time of data collection, or—in the case of sharing existing data—before making data available to other teams, can therefore save time and efforts, improve scientific value and help to prevent erroneous assumptions and biased results.[4]

The current trend of data sharing and open access, often involving large datasets from different countries, increases the risk of incorrect handling of missing data since there is no link between the researchers performing the collection and those analyzing the data. To the best of our knowledge, there are no clear methods for conveying the reasons for missing data, despite a large body of literature on how to prevent and analyze missing data.[5] Therefore, we developed a list of context-free codes of missing data and used them in a project to pool three existing datasets from three countries as well as for a new, international randomized controlled clinical trial[6].

## Materials and methods

The ‘Prevention of dementia by intensive vascular care (PreDIVA, ISRCTN 29711771)’, ‘Finnish Geriatric Intervention Study to Prevent Cognitive Impairment and Disability (FINGER, NCT 01041989)’ and the ‘Multidomain Alzheimer Preventive Trial (MAPT, NCT 00672685)’ are recently completed large randomized controlled clinical trials with a total of over 6400 participants.[7–9] The ‘Healthy Ageing Through Internet Counselling in the Elderly (HATICE) randomized controlled clinical trial’ is an ongoing study on the effect of a multidomain internet intervention on cardiovascular risk factors, in over 2700 participants in three countries.[6]

To identify missing values, all variables in the three completed trials[7–9] were evaluated by an international team collaborating in the HATICE consortium, consisting of clinical researchers and epidemiologists with extended experience in designing and conducting trials and an Information System expert. The individual research teams of each trial first listed all situations that led to missing values in their study. Next, the Information System expert merged all missing situations into one list of missing-categories (S1 File). In a consensus meeting, the international team agreed on the most important missing-categories, taking into account their external applicability. For pooling datasets, an additional missing data code was created for variables that were not collected by at least one of the other studies. We used numerical codes to accommodate analyses in most statistical packages. To avoid confusing missing data with non-missing data we used codes with 6 digits and starting with ‘9’; e.g. 930000 for ‘not applicable’ (NA) and 931000 for ‘not applicable due to conditional value’ (NAC). Before pooling data from the three trials in an online platform specifically designed for the purpose, we converted the original missing data encoding of every dataset into the encodings represented in Table 1. To establish the applicability of the encodings on a trial that was not used to develop the encodings, they were implemented at the data collection stage of the currently ongoing HATICE trial[6].

**Table 1:** Categories of missing data.

Source	Category of missing data	Examples	Abbreviation	Type_a_
Participant and/ or participation characteristic	Assessed but the participant does not know	Family history could not be answered, because of broken contact	ASSU	MCAR
	Assessed but the participant was not able to provide the information	Disability preventing a physical test	ASSD	MNAR_c_
	Refusal	Participant does not want to tell his weight because he is embarrassed	ASSR	MNAR
	Not applicable_b_	“Do you feel disabled in doing volunteer work” in case the participant is not engaged in any volunteering	NA	MNAR_c_
	The visit has been missed	In case of a missed visit, all variables for this visit are missing	MISS	MAR/ MNAR_d_
	Dropout	In case of dropout, variables subsequent to the date of dropout are missing	DROP	MAR/ MNAR_d_
By design	Not assessed, variable not in the study	Only applicable for data pooling	NASS	MCAR
	Not applicable because of conditional variable_b_	Date of birth of siblings if participant does not have siblings _e_	NAC	MNAR_c_
	Due to random subsampling	Expensive measurement only performed in random subsample. For others these values are missing.	RS	MCAR
	Answer/value not available yet	Blood sample was collected though not analyzed yet	NAV	MCAR
Procedural error	Not assessed/ registered, by mistake	Box of questionnaires got lost	ERR	MCAR

_a_ MCAR: missing completely at random, MAR: missing at random or MNAR: missing not at random. The types are an indication of the most common scenarios fitting to this category (see Discussion section).

_b_ Often, the question whether it is applicable (for instance ‘do you take medication’) is not included. In this case NA has to be filled in manually. However, if this question is asked and therefore the conditional variables can be skipped, a digital questionnaire can fill out the NAC category automatically.

_c_ In ‘ASSD’ and ‘NA’ categories the fact that the value is missing depends on the reason why it is missing, so this fits the definition of MNAR. However, how to handle this in the analyses should be decided on a case to case basis.

_d_ We advise for these categories to make subcategories, specific to the study (see Discussion section).

_e_ In a digital questionnaire it is possible that the conditional questions are automatically skipped so the participant does not have to deal with the questions that are not applicable to their situation. To inform the data analyst that the variable is deliberately skipped the NAC value will be automatically filled out.

Technical details on the context-free data encoding have been published previously (S2 File).[4]

## Results

We identified 11 different types of missing data (Table 1). These can be divided into the following categories: missing due to participant and/or participation characteristics (n = 6), missing by design (n = 4), and due a procedural error (n = 1). The 11 missing encodings were sufficient to recode all missing data in the three completed trials[7–9] and the HATICE trial.[6]

## Discussion

To initiate a systematic approach for context-free missing data encoding, we described 11 separate missing codes that could be classified in three categories: missing due to participant and/or participation characteristics (n = 6), missing by design (n = 4), and due a procedural error (n = 1).

Clearly, a careful balance is needed between accuracy (determined by the number of missing data categories) and the validity of the information. Consequently, the missing data categories that we identified, cannot be used one on one to determine whether data are MCAR, MAR or MNAR. For instance, in the ASSU category (asked but participant does not know the answer) not knowing the answer could be independent of observable and unobservable parameters of interest, and as such be MCAR. However, if the outcome is cognitive function, not knowing is probably informative and MNAR applies. To account for all possible scenarios, the categories may need to be further subdivided. However, too many missing data categories may be confusing for the person filling out the assessments, particularly if this person is a participant. This may jeopardize the validity of the information. The missing data encoding cannot cover the nuances that can be explained in free text. Missing data encoding and free text can co-exist. Especially in big studies, free texts are difficult to take into account and the missing encodings have most of their value.

For the MISS (visit missed) and DROP (dropout) categories, which are generally filled out by the researchers, subcategories are recommended. Current common practice is to have a separate variable for reasons for dropout which can be combined with the system missing variables to decide on analytical techniques. One could choose to integrate the reasons for dropout (or missed visits) in the missing encodings. This would require the MISS (visit missed) and DROP (dropout) categories to be divided into subcategories, specific to the study. For instance a code 911000 for dropout because deceased, a code 912000 for dropout because of adverse effects of treatment, etc. As these categories are registered already in most studies, no further confusion is expected from this approach.

A major strength of our approach is the combination of expertise from information specialists, clinical researchers and epidemiologists. Both from an information systems perspective and an epidemiological perspective, our efforts can be a starting point for adopting these encodings as well as further developing categories applicable to specific situations/ domains. Current existing standard classifications/ nomenclatures/ terminologies are lacking a system for missing data encoding. Our encodings can, for instance, easily be adopted in existing standard Case Report Forms such as those in CDASH (Clinical Data Acquisition Standards Harmonization) of CDISC (Clinical Data Interchange Standards Consortium) thereby contributing to their mission to enable data sharing[10]. The issue of missing data is relevant for all domains using data intensively. Our work has focused on healthcare-related research, but can be applied to other branches of research, after appropriate validation. When different studies apply the same missing encodings, recoding for data pooling will be reduced in the future. Whether a higher level of granularity in missing encodings can prevent biased results, loss of power and reduced generalizability will have to be further investigated.

## Conclusions

Missing data can rarely be fully avoided, but not knowing why data are missing can be avoided. Capturing information on the reason for missing data values at the moment of data collection reduces the loss of relevant information and thereby the need for assumptions in the analysis phase. Broad implementation of context-free missing data encoding may enhance the possibilities of data sharing and pooling, thus allowing more powerful analyses using existing data.

## Supporting information

S1 File Missing data encodings in dataset. (CSV)

S2 File Conference proceeding on missing data encodings for information specialists. Meiller Y, Guillemont J, Beishuizen CR, Richard E, Kivipelto M, Andrieu S. An IS Approach for Handling Missing Data in Collaborative Medical Research. Twenty-second Americas Conference on Information Systems; San Diego2016.
(PDF)
