# Supplementary material for: Improving data sharing in research with context-free encoded missing data
Source: PLoS One. 2017 Sep 12;12(9):e0182362. doi: 10.1371/journal.pone.0182362 (PMC5595279; doi:10.1371/journal.pone.0182362)
Supplement: S2 File — Meiller Y, Guillemont J, Beishuizen CR, Richard E, Kivipelto M, Andrieu S. An IS Approach for Handling Missing Data in Collaborative Medical Research. Twenty-second Americas Conference on Information Systems; San Diego2016. (PDF) [file pone.0182362.s002.pdf]

ResearchGate

See discussions, stats, and author profiles for this publication at: https://www.researchgate.net/publication/304024535

An IS Approach for Handling Missing Data in Collaborative Medical Research

Conference Paper · August 2016

CITATIONS
0

READS
60

6 authors, including:

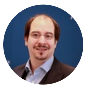

Yannick Meiller
ESCP Europe
19 PUBLICATIONS **60** CITATIONS
SEE PROFILE

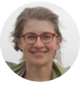

Cathrien Beishuizen
Academisch Medisch Centrum Universiteit van …
8 PUBLICATIONS **22** CITATIONS
SEE PROFILE

All content following this page was uploaded by Yannick Meiller on 17 June 2016.

The user has requested enhancement of the downloaded file. All in-text references underlined in blue are added to the original document and are linked to publications on ResearchGate, letting you access and read them immediately.

An IS Approach for Handling Missing Data in Collaborative Medical Research

Full paper

Yannick Meiller[1], Juliette Guillemont[2,3], Cathrien Beishuizen[4], Edo Richard[4,5], Sandrine Andrieu[3], Miia Kivilpeto[6, 7]

1- ESCP Europe, Paris, France
2- Novapten, Paris, France
3- INSERM, University of Toulouse UMR1027, Toulouse, France
4- Department of Neurology, Academic Medical Center, University of Amsterdam, Amsterdam, The Netherlands
5- Department of Neurology, Radboud University Medical Center, Nijmegen, The Netherlands
6- Aging Research Center, Karolinska Institutet/Stockholm University, Stockholm, Sweden
7- Institute of Clinical Medicine/Neurology, University of Eastern Finland, Kuopio, Finland

Abstract

As more and more data are shared, the possible distance between data collection and data analysis has increased. This makes missing data handling more difficult, because of the possible loss of information between collection and analysis. We wondered how information about missing data could be shared in order to improve missing data handling. No answer could be found in the literature. Therefore, we conducted an empirical study over three large medical datasets. We observed a diversity of practices and opportunities to improve them. We designed a way of transmitting information about missing data, easy to implement, based on what we empirically learned. Our propositions have been implemented in a large scale medical research project, giving the opportunity of a second empirical study for future works.

Keywords: missing data, collaboration, data sharing, representation.

Introduction

With the development of data sharing in healthcare and medical research (including open data), the issue of missing data is growing. Indeed, when getting data collected by others, it is important to be aware that data are missing, and in order to be able to handle them we need to know more about these missing data.

Our work focuses on missing data, and more precisely on the transmission of relevant information about missing data from the data collection stage to the data analysis stage. This transmission is crucial for improving the analysis of missing data and for improving data sharing as well as data reuse. Our work positions missing data in an Information System perspective.

In (NRC 2010), the authors state that the Federal Drug Administration (FDA) has identified the issue of missing data as a priority topic. They also state that the treatment of missing data in clinical trials should have a higher priority for sponsors of statistical research, such as the National Institute of Health and the National Science Foundation. Indeed, in healthcare-related studies, quantitative data analysis is central and the question of missing data is particularly acute (both for its impact on analysis and for its variety of causes, including dropouts in clinical trials).

Missing data are crucial because they impact the analysis of the whole dataset. Indeed, these missing data are not void only. They are a part of the dataset which we do not know. This part of the dataset has some meaning, but we cannot access it. The very fact it is missing may be meaningful in itself. We cannot only reshape the dataset, keeping data we have and discarding missing data. Doing so could bias strongly the results of any subsequent analysis.

The rest of this paper will continue as follows. First, we position our work in the Information system field of research. Secondly, based on existing literature, we go into more details about what are missing data and how they can be handled. We show that the literature does not say much about transmitting information about missing data. Then, in the third section, we present an empirical study, based on three

real large datasets and an initiative for pooling them in a fourth dataset. We will empirically show, with these examples, that the current practices do not complement the void left by literature – thus opening an avenue of research for the IS community. In the fourth section, we present propositions for improving missing data handling. Finally, in the fifth section, we describe the implementation of our propositions in a large-scale project, starting a second empirical study, and discuss future work.

How does our work relate to information systems?

Without denying that multiple ways of defining information systems (IS) have been proposed (Alter 2013; Neufeld et al. 2007; Westfall 2012), we can admit that most of them consider that an IS deals with information, technologies, processes (organization), and persons.

The object of our work is at the core of IS: we consider data which are collected by a dedicated team, possibly using information technologies, and then analyzed by a dedicated team (certainly using information technologies), in order to make a decision. Thus we have a collection stage, an analysis stage and a decision stage, as well as people and technologies. Information is processed at every stage. There may be a long time between collection and analysis (often due to the duration of the clinical trial). A unique team may be in charge of the whole process, or different teams may be involved. In the latter case, explicit transmission of information from the collection team to the analysis team is involved. Both these teams have to be connected. They can work for the same entity or for different entities. The focus of our work is exactly there: what is transmitted about missing data? Is this sufficient at analysis time? Could we improve the analysis by transmitting more information?

As will be shown in the literature review, issues raised by missing data have been tackled already. However, stages of the process are considered independently from each other: methods for decreasing the probability of missing data during the collection stage are proposed or statistical methods to handle missing data, during the analysis stage. However, the connection between these stages has not been addressed. On the contrary, we propose to consider the whole system, with information flowing from the collection stage to the analysis stage. Doing so, we adopt an IS approach to missing data handling.

We believe this approach is all the more relevant as current trends such as data sharing and open data make it likely to have different teams (possibly not knowing each other) in charge of the collection stage and of the analysis stage. We think sharing what is missing is necessary to increase data sharing in general.

Missing data in the literature

Reasons why data are missing

This is common to have data missing in a dataset (DeSouza et al. 2009; Jackson et al. 2010; Karanja et al. 2013; NRC 2010; Walton 2009). There are many reasons for data being missing.

Data can be lost after collection: destruction of data (hardware failure, fire, flooding, theft… connected with poor backup), incapacity to retrieve old data, mistakes during copy or transcription, etc.

Most often, data are missing because they could not be collected properly. In some cases, collection is initiated but cannot be carried out successfully. Suppose for instance that blood samples are collected from people involved in the study, in order to analyze them. These analyses will provide data for the study's dataset. Once the sample collected, it can be lost or it can be unusable for the analysis (not enough material, damaged material, specificities incompatible with the analysis method…).

In some other cases – probably most often – data are just not collected: the question has not been asked, or the subject did not answer. The subject did not answer because he did not want to, or because he could not. Here, the design of the questionnaire or of the case report form plays a central role: for example, is it possible to report that the subject could not answer because of some physical impairment?

In this category falls the main reason for missing data in datasets related to longitudinal study such as clinical trials: dropouts. Between the beginning of the study and the end of the study, it is likely that part of the studied population is going to drop out – i.e. to exit the study. Finally, some data may be missing by design. For example, some questions are conditional: depending on what is answered question 1, the next question will be question 2 or question 3. Therefore, for some subjects, the final dataset will have no data for question 2. This is "normal". However, when considering the dataset we have to remember the conditional structure of the questionnaire – otherwise these data are as missing as any other missing data.

Missing Data Classifications

The most widely used missing data classification is related to the relationships between variables and the probability of data being missing. It distinguishes data missing completely at random (MCAR), missing at random (MAR), and missing not at random (MNAR) (Little and Rubin 2002; Rubin 1976).

Missing data are MCAR if the fact they are missing is totally independent of all variables included in the study. For example, consider the study of the dietary habits of people living in France over a period of time of five years. Suppose after two years a subject moves to the Netherlands because her employer offers her a new position there. Her moving to the Netherlands induces missing data (for the three remaining years before the end of the study) but has nothing to do with her dietary habits.

Missing data are MAR if they depend on other variables of the dataset but do not depend on the missing data themselves. This is the case for conditional questions or for conditional situations. Consider for example a medical study in which, first the breathing capacity of the subject is measured, and, only if this capacity does not reach some threshold, then additional examination is conducted (such as X-ray imaging of the lungs for instance). For a given subject, missing data about the X-ray imaging is totally dependent on the evaluation of the breathing capacity.

Missing data are MNAR if they depend on what would have been the data if they were not missing. For example, when a questionnaire asks people their habits regarding alcohol consumption, the ones with heavy alcohol consumption may prefer not to answer the question. Here missing data can say something about what has not been said.

Focusing on dropouts in clinical trials, (NRC 2010; Wei 2011) choose another perspective and propose to distinguish intrinsic and extrinsic dropouts. The former ones are directly related to what is tested during the study. In the context of a clinical trial, intrinsic dropouts are directly related to the treatment being tested: people dropped out because of secondary effects, because the pain was too high, etc. Extrinsic dropouts are related to causes which are not connected to the treatment (even though they may be linked to the study): for example, they moved to another city, where the treatment cannot be administered. They argue that intrinsic dropouts should be included somehow in the population taken into account when statistically analyzing the dataset, since these missing data say something about the treatment being tested. This is a kind of situation for which discarding missing data could bias the results.

Handling missing data at analysis time

Once facing a dataset with missing data, statistics offer a variety of methods to handle them. Some of them are quite basic: listwise deletion, pairwise deletion, missing data replacement by one given value extrapolated from the rest of the dataset.

These methods may reduce too heavily the size of the sample or may simply lead to strong biases. It is now generally recommended not to use these methods (Baraldi and Enders 2010; Walton 2009; Wittes 2009).

More sophisticated methods exist and are preferred by methodologists (Baraldi and Enders 2010; Karanja et al. 2013; NRC 2010). Among them maximum likelihood, and multiple imputation seem to be quite popular. We can also cite Bayesian methods, expectation maximization and generalized estimated equations… Detailing these methods would be beyond the scope of this paper.

The main point to keep in mind here is that the choice of methods to apply relies partly on the type of missing data, namely whether they are MCAR, MAR or MNAR. If one doesn't know the type of missing data he is facing, he cannot determine which method is the most suitable. However, a major issue is that with the dataset only it is very difficult – if not impossible – to know in which category fall missing data. (Gewandter et al. 2014; NRC 2010) talk about "untestable assumptions".

Missing literature

The issue of missing data has attracted a growing interest in the literature, in particular in the field of medical research, mainly as a question of statistics. On the one hand, papers focus on the analysis stage – assuming one faces a dataset with missing data and studying how to handle them. On the other hand, papers focus on the collection stage, aiming at finding a better study design and a better study implementation in order to minimize the amount of missing data. However, none tackles the information system handling data, and therefore missing data, as a whole. To our knowledge, there is nothing about

what information should be transmitted, and how it should, from the collection team to the analysis team in order to improve missing data handling, and therefore improve the analysis of the dataset.

There is room for multiple practices, which is likely to cause major problems when sharing datasets. The situation could be particularly difficult with open data since there is no link between the team in charge of the data collection and the teams which will download and analyze the dataset. Similar difficulties are likely to arise when data previously collected in a given context are reused in another context.

Maybe solutions come from practice. In real datasets, how missing data are represented? What does happen when data are shared among multiple teams? Is there a common practice?

Empirical study

Our study of empirical data aims at two objectives:

1. Exploring the reality of practices concerning missing data handling;
2. Gathering elements to help – if needed – design methods for improving this handling of missing data.

Indeed, on the one hand, our literature review showed that data themselves are likely not to say enough to determine the type of missing data and therefore to determine which statistical method(s) would be the most suitable. On the other hand, our literature review showed nothing is said about how information about missing data could be or should be transmitted from the collection stage to the analysis stage.

However, in medical research datasets with missing data are commonly processed. Therefore, by studying empirical data we want to gauge the reality of missing data and how practitioners actually handle them. In particular, we wonder whether some sort of common practice has emerged.

By doing so, we also gather characteristics of missing data. These could help us improve their processing.

Methodology

In order to answer our questions, we decided to work on datasets themselves, and complementing this work with interactions with the teams responsible for theses datasets. We focused on a European collaborative medical research project. This project is named HATICE (Healthy Ageing Through Internet Counselling in the Elderly) (Mangialasche et al. 2013; Richard et al. 2016). The aim of the Hatice project is to study whether an interactive Internet platform with coaching for self-management of cardiovascular risk factors can improve the cardiovascular risk profile of older people and reduce the risk of cardiovascular disease and cognitive decline.

This project is particularly interesting for our work on missing data because:

- Three of the teams contributing to the project had already conducted large randomized clinical trials on similar subjects. These studies have been designed prior to the idea of setting up the Hatice project. Thus, each team has designed its own study, with its own data collection strategy and its own encoding for it – including its own way of handling missing data. This allows the comparison of three approaches in a similar intellectual context.
- The Hatice project includes a step for pooling all these data together, and analyzing them, in order to take advantage of these data when designing the Hatice randomized clinical trial (Richard 2014). This gave us the opportunity to study missing data in a situation where data had to be shared.

Original datasets

The three datasets at hand have been generated by the following research projects:

- Finnish Geriatric Intervention Study to Prevent Cognitive Impairment and Disability (FINGER) – led by the University of Eastern Finland and the Aging Research Center – ARC (Karolinska Institute and Stockholm University - Sweden);
- Multidomain Alzheimer Preventive Trial (MAPT) – led by INSERM U1027 (INSERM and University of Toulouse III - France);
- Prevention of Dementia by Intensive Vascular Care (PreDIVA) – led by the Academic Medical Center of Amsterdam – the Netherlands.

FINGER (Kivipelto et al. 2013) is a medical randomized controlled trial designed to delay cognitive impairment among high-risk 60-77 years old individuals. The population of the study is randomized into two groups to receive either a multi-domain intervention (nutritional guidance, exercise, cognitive training, social activities, intensive monitoring and management of vascular risk factors) or regular health advice. It gathers about 1000 participants, in Finland. The dataset is structured in 131 variables.

MAPT (Gillette-Guyonnet et al. 2009) is a medical randomized controlled trial aiming at evaluating the efficacy of a multidomain intervention (nutritional, physical and cognitive training) and omega 3 treatment in the prevention of cognitive decline in frail elderly persons aged 70 years or over. It has also collected imaging and biological data which could be used for future prevention and treatment trials. MAPT has gathered about 1200 participants, in seven French cities, for a three-year-long study. The dataset is structured in about 845 variables

PreDIVA (Richard et al. 2009) is a medical randomized controlled trial which aim is to assess whether nurse-led intensive vascular care in a primary care setting decreases the incidence of dementia and reduces disability. It focuses on both lifestyle interventions and medical interventions based on management of blood pressure, cholesterol levels, smoking habits, body weight, and level of exercise. It has gathered 3526 participants (between 70 and 78 years old) for a 6-to-8-year-long follow-up. The dataset is structured in about 1222 variables.

How are missing data represented?

Studying these three large datasets, we found that missing data are encoded differently in the three datasets, some encodings are contextual, the typologies of missing data are different and part of the missing data typology is rebuilt at analysis time, from existing data and extra information.

Different encodings for missing data

Here are the different encodings. All the datasets use the character used by the system to indicate a value is missing (here the character coma ',' or the character dash '-'). One dataset uses also the digit'0'. Another dataset uses the digit '2'. The last dataset uses six different numbers: -1, 3, 4, 9, 88 and 99.

The three teams prefer a numeric encoding (except for the system character). Firstly, they are used to it: in these studies, all data are encoded through numbers. Secondly, the statistical software they use facilitates this type of encoding by letting the user attach a label to each possible numerical value. For instance, one can specify that variable 1 will have as value 0 or 1, the label "female" being attached to 0 and the label "male" being attached to 1.

Some encodings are contextual

A given value representing missing data can have different meanings in the same dataset, depending on the variable at hand, and possibly depending on the values of some other variables. For example, in the third study, '3' can be used to encode data missing because the related question was not applicable or because the question was asked (therefore being applicable) but the subject did not know the answer.

On the contrary, different codes can be used for the same meaning. In the same dataset, '3' and '4' are both used to encode non-applicable questions.

Codes are not assigned to types of missing data for a whole dataset. The way of interpreting the meaning of a code depends on the variable being considered. This is what we call a contextual encoding.

Part of the missing data typology is rebuilt at analysis time

The dataset itself is not self-sufficient to grasp the whole semantic structure of the missing data typology. Of course, the data dictionary helps to understand the meaning of each code for a given variable (context). However, this is still not sufficient to grasp all the different types of missing data which can be distinguished in the data set.

Part of the typology has to be rebuilt, using extra knowledge about the study design. For example, in all three studies, conditional questions are handled this way. A "conditional question" may not be asked depending on the answers collected for previous questions. In the dataset, if there is no answer to the second question, then, depending on the value previous variables, either data are missing because the

question was not applicable or data are missing for another reason. Note that in this case we do not know this reason (we forgot to ask the question, the subject did not remember, or did not want to answer, etc.).

Note that knowing whether a question is conditional requires knowing the study design. If the same team designs the study, collects data and analyzes them, there is likely to be no issue. If a different team is in charge of data analysis, then information about the study design has to be transmitted in addition to the dataset and the data dictionary.

Different typologies of missing data

The semantic granularity, i.e. the number of types of missing data which are distinguished, is different from study to study. Because of the contextual encoding, it cannot be derived directly from the number of codes. In our example, both studies using two different codes for missing data are able to distinguish three types of missing data. The study using seven different codes distinguishes four types of missing data. In both situations we take into account all types of missing data which can be identified, also using additional information about the study design. Note that having access to the dataset (and the attached data dictionary) does not provide this extra information.

Only two types of missing data are common to all three datasets: data are missing for no specified reason or data are missing because of a conditional question structure. Three extra types of missing data can be identified in these datasets: data are missing because the question was not applicable (in one dataset), because the respondent did not know the answer (in one dataset), or because the respondent refused to answer (in two datasets).

Impact of pooling data on missing data

When pooling data, missing data are also pooled together. A common data dictionary has to be built from the original data dictionaries, and data have to be recoded accordingly. This also applies to missing data.

Either all semantics are flattened by encoding missing data into “missing”, with no particular distinction, or we try to keep the semantics which were included in the original datasets. The latter is obviously preferred, but requires rebuilding the type of missing using extra knowledge about each study design. Indeed, once data are pooled together, it would be difficult to know that two missing values have to be interpreted differently, because they come from different original studies and therefore correspond to different extra knowledge. Note once again, that this extra knowledge about original studies is likely not to be available to users of the pooled dataset.

Moreover, the process of pooling data together can by itself generate missing data. In our empirical field, all three studies have been designed independently, with no perspective of being pooled together. In fact, we would be in a similar situation when considering exploiting open data from diverse sources. As a consequence, a given question or measure can be part of all studies, of none of them or of some of them. If you decide to keep only variables included in all studies, you may end up with a very small group of variables. In the Hatice project it has been decided to pool together all variables included in at least two studies out of three. As a consequence, for a given variable included in two datasets only, in the final (pooled) dataset data will be missing for all the population of the third original dataset. It can be relevant to know that these data are missing due to the pooling process, and for no other reason which may be linked to the original data collection. This is all the more important as the final dataset is proposed for sharing, by itself, without the original datasets.

Our Proposal

In our empirical study, the dataset (and its data dictionary) are never self-sufficient to determine the category of missing data. Part of the process of categorizing missing data requires deducing categories from data and extra knowledge about the study. This has two major drawbacks: this is time consuming and this requires extra knowledge. Both impact negatively data analysis productivity and are likely to impact negatively data sharing.

Moreover, a few categories of missing data are distinguished. This includes the generic category “missing” with no specified reason. Therefore, we still have a large loss of information between collection time and analysis time. This information would be useful to improve how missing data are processed.

Here are some propositions to reduce this loss of information.

General architecture

In a dataset, we propose to link each variable V_i to a metadata variable MV_i so that when data are missing in V_i, MV_i indicates the type of missing data. The idea behind this is to include within the dataset information about the missing data. Furthermore, we propose the encoding of metadata about missing data to be context-free. This aims at making it straightforward how to recognize which type of missing data is being faced, without rebuilding categories with extra knowledge. Note that such a context-free encoding of missing data categories would also facilitate the use of automatic processing – for example when recoding data or when pooling datasets together.

With such architecture we reduce the amount of information lost between the data collection stage and the data analysis stage. By making available a dataset, and its data dictionary, we make available at the same time information about missing data.

Proposition of a Set of Categories

In table1, we propose a set of nine categories for missing data and one subcategory. These are the possible values for the metadata variables MV_i. The first column lists the different types of missing data we propose. The second column presents a short description. The third column provides examples of possible use.

Type	Explanation	Example
NASK	The question was not asked in the particular study, therefore the variable does not exist in that study (only applicable for data pooling)	
ERR	A question has not been asked by the assessor by mistake and should have been asked.	A problem occurs with a blood sample, making it unusable.
ASKU	A question has been asked, but the participant does not know the answer	It is asked whether any ascendant of the subject has or has had dementia, but the subject does not know.
ASKD	The question was asked but the participant was not able to answer	The participant has some disability preventing him from answering.
ASKR	The participant refuses to answer.	
NA	A question refers to a situation that is not applicable to the participant.	It is asked how many types of medications the participant has been prescribed, but he has no medication prescribed.
NAC	A sub-category of NA. The question is not applicable because of conditional variables. If a sub-question is asked after a question that was not applicable to the participant.	It is asked if the participant uses the medication as prescribed by the doctor. This is not applicable if the participant doesn't use any medication in the first place.
NAV	The answer/value is not yet available.	A collected blood sample has not been analyzed yet.
MISS	In case the participant did not show up for a study visit, all variables for this visit are MISS.	
SYSMIS	The reason why data are missing is unknown (default category).	

Table 1. Proposition of a set of missing data categories

We designed this typology by including the five categories encountered in individual datasets during our empirical study (here: ASKU, ASKR, NA, NAC and SYSMIS). We also included the category generated by pooling data together (here below: NASK). Then, by brainstorming with practitioners in charge of the datasets, we added three other possible categories of missing data: NAV, MISS and ERR.

Note that NAC is a subcategory of NA, opening the possibility of a hierarchical set of categories. Missing data of type NAC played an important role in the data sets we have studied in our empirical study. Indeed, it is quite common to have questions or measurements which are conditioned by previous questions or measurements. Nevertheless, these missing data "by design" were not identified as such, forcing the data analysts to exploit extra knowledge about the study design in order to know why these data were missing.

ASKU, ASKD and ASKR are subject centered. They allow making the difference between not being able – literally - to answer the question (ASKD), not having the knowledge necessary to answer the question (ASKU) or not wanting to answer the question (ASKR). According to practitioners, these distinctions are likely to be meaningful in themselves.

Expected Impact on Missing Data Handling

This representation of missing data enables to include more information about missing data within the dataset itself. This information is mainly focused on the reasons why some data are missing. Our idea is to reduce the loss of relevant information between data collection and data analysis and therefore improve the possibilities of data processing for missing data and improve the reusability of the dataset.

This typology does not implement the common MCAR-MAR-MNAR typology for handling missing data. However, we expect it to give more information when it comes to process missing data in order to decide whether we are facing MCAR, MAR or MNAR data, and hence choose an appropriate method.

For example, data missing because errors have been committed (type ERR) are MCAR. In a pooled dataset, NASK missing data (data missing because the related variables did not exist in one of the original datasets which have been pooled together), are MCAR also – or MAR if the pooled dataset contains a variable identifying the original dataset.

Knowing whether a question has not been answered because the participant did not know the answer, was not able to answer because of some impairment or clearly refused to answer provides much more information to decide whether we are facing MCAR, MAR or MNAR missing data than just "missing…".

On the other side, the risk is greater that the higher number of categories jeopardizes the quality of data collection when it comes to encoding missing data.

Implementation

First, the missing data categories which we propose have been used when pooling data from the three studies, as part of the Hatice project. Thus, we converted the original missing data encoding of every dataset (which was contextual and requiring extra knowledge) into a common, context free, self-sufficient encoding. With this recoding, the final (pooled) dataset can be transmitted to other teams more easily (without extra knowledge about the study design). This first implementation showed us that this conversion did not give rise to major issues. However, as our typology has been designed based on the study of these three datasets, this was expectable.

Second, the Hatice randomized clinical trial has started collecting data, in three countries. The architecture and the missing data categories we propose have been implemented from the beginning: impacting database structures, online questionnaires and interfaces for collecting data.

The collection stage is ongoing. So far 686 variables are included in data collection and 728 642 elements of data have been collected (including missing data). Already, all categories of missing data but one have been used. The unused category of missing data is NASK (Variable not in the study) which is specific to data pooling. Hence, it is normal this category remains unused.

As could also be expected, the frequency of use is different from category to category. A first observation had been carried out at the very beginning of the data collection – "only" 95 289 elements of data had been collected already. Only 4 specific categories of missing had been used: NA (not applicable), NAC (not applicable because of conditional variable), NAV (answer/value not yet available) and ASKU (asked but the participant does not know the answer). The unused categories appeared later, while the data sample was growing- including rarer cases. For example, as the Hatice project is a clinical trial, all participants are volunteers. Therefore, we can expect that they will rarely refuse to answer a question. This would probably be very different in a survey targeting people randomly.

Thus, the data collection teams use different categories of missing data with no identified difficulties. Better, in this project, we noticed that observing which codes are used by the different teams for missing data can contribute to insure cohesion throughout the trial and to discuss how data are collected by the different teams (in different countries).

An interesting configuration is when several categories of missing data appear for the same variable. For example, a question in the Hatice project is about the medical history of the respondent's siblings. For this variable, data are missing for part of the respondents. Missing data fall either in category NA (not applicable) – because the respondent does not have any siblings, or in category ASKU (the participant does not know the answer). This difference is meaningful. Just with the missing data categories, we know whether further investigation is possible (asking the respondent to ask his/her siblings for instance) or not

(if the respondent does not have any sibling). Thus, the dataset being collected already conveys more information about missing data than it would have without these context-free codes for missing data.

Conclusion and future work

Sharing data often implies there will be teams analyzing data whereas they neither contributed to the collection of these data, nor to the design of this data collection. If we consider globally the IS behind data processing, data sharing adds more actors and it impacts processes. It makes explicit the transmission of information from collecting teams to analysis teams.

In the literature, we found nothing about transmission of information about missing data. Nevertheless, the literature shows that processing properly missing data is a crucial issue and that this can be complex. Moreover, the literature shows that it can be very difficult to process correctly missing data because of a lack of information about the type of missing data being faced.

Finding no solution in the literature, we turned towards medical research practitioners. We completed an empirical study on three major datasets, from three different medical studies in similar areas of interest, studying missing data in each of them and when pooling them together. Our first contribution was to show the following results: there was no common typology of missing data; encoding of missing data was contextual – i.e. it depends on the variable at hand; determining the type of missing data required additional knowledge about the study design; a few types of missing data were distinguished; pooling data together can generate missing data.

Surprisingly, both our literature review and our empirical study show that little attention has been paid to the development of efficient methodologies for transmitting information about missing data, despite the fact processing missing data is considered as an important issue. This could be due to the fact this paradox is less acute when a same team is in charge of collecting data and analyzing them – i.e. as long as data are not shared (and therefore there is less need for an IS to connect people).

Our results open a new avenue of research: bringing an IS approach to the processing of missing data. We contribute to it by proposing a way to reduce the loss of information about missing data between the collection stage and the analysis stage. We implemented our proposition in a large medical project including data collection and data analysis, and involving several teams in different countries. There has been no difficulty to use our categories and the dataset is richer in information about missing data.

Our work has focused on healthcare-related research, but it is relevant for other domains using data intensively – therefore facing the issue of missing data.The implementation of our approach as part of the Hatice project, as well as other potential field studies, will contribute to our future works along three axes: discuss the design of the missing data categories; quantify empirically how the missing data categories we propose improve missing data processing during the analysis stage; evaluate the impact of our richer representation of missing data on data-sharing.

Acknowledgements

HATICE (www.hatice.eu) is a collaborative project co-funded by the European Union's Seventh Framework Program (FP7, 2007-2013), under grant agreement No 305374.

References

Alter, S. 2013. "An' Interpretary' for the IS Discipline, a Compendium of Interpretations of Basic IS Concepts and Methods from Different Theoretical Perspectives," *Communications of the AIS* (33).

Baraldi, A. N., and Enders, C. K. 2010. "An introduction to modern missing data analyses," *Journal of School Psychology* (48:1), pp. 5–37.

DeSouza, C., Legedza, A., and Sankoh, A. 2009. "An Overview of Practical Approaches for Handling Missing Data in Clinical Trials," *Journal of Biopharmaceutical Statistics* (19:6), pp. 1055–1073.

Gewandter, J., McDermott, M., McKeown, A., Smith, S., Williams, M., Farrar, J., Turk, D., and Dworkin, R. 2014. "Reporting of missing data and methods used to accommodate them in recent analgesic clinical trials: ACTION systematic review and recommendations," *PAIN* (155:9), pp. 1871–1877 .

Gillette-Guyonnet, S., Andrieu, S., Dantoine, T., Dartigues, J.-F., Touchon, J., and Vellas, B. 2009. "The Multidomain Alzheimer Preventive Trial (MAPT): A new approach to the prevention of Alzheimer's disease," *Alzheimer's & Dementia: The Journal of the Alzheimer's Association* (5:2), pp. 114–121.

Jackson, D., White, R., and Leese, M. 2010. "How much can we learn about missing data?: an exploration of a clinical trial in psychiatry," *Journal of the Royal Statistical Society: Series A* (173:3), pp. 593–612.

Karanja, E., Zaveri, J., and Ahmed, A. 2013. "How do MIS researchers handle missing data in survey-based research: A content analysis approach," *Internat. Journal of Inform. Mgt*(33:5), pp. 734–751.

Kivipelto, M., Solomon, A., Ahtiluoto, S., Ngandu, T., Lehtisalo, J., Antikainen, R., Bäckman, L., Hänninen, T., Jula, A., Laatikainen, T., Lindström, J., Mangialasche, F., Nissinen, A., Paajanen, T., Pajala, S., Peltonen, M., Rauramaa, R., Stigsdotter-Neely, A., Strandberg, T., Tuomilehto, J., and Soininen, H. 2013. "The Finnish Geriatric Intervention Study to Prevent Cognitive Impairment and Disability (FINGER): Study design and progress," *Alzheimer's & Dementia* (9:6), pp. 657–665.

Little, R., and Rubin, D. 2002. *Statistical Analysis with Missing Data*, Wiley.

Mangialasche, F., Kivipelto, M., Andrieu, S., Coley, N., Ngandu, T., Moll van Charante, E., Brayne, C., Meiller, Y., Van Groep, B., Soininen, H., Van Gool, W., and Richard, E. 2013. "Use of new technology to improve dementia prevention: The HATICE project," *Alzheimer's & Dementia* (9:4), p. 881.

National Research Council (NRC) 2010. *The Prevention and Treatment of Missing Data in Clinical Trials* (The National Academies Press.), Washington DC - USA: National Research Council.

Neufeld, D., Fang, Y., and Huff, S. 2007. "The IS Identity Crisis," *Communications of the AIS* (19).

Richard, E. 2014. "Towards an internet-intervention for prevention of stroke, cardiovascular disease and dementia: sharing data to improve trial design - the HATICE project," Europ. Stroke Conf. Nice Fr.

Richard, E., den Heuvel, E., Moll van Charante, E., Achthoven, L., Vermeulen, M., Bindels, P., and Van Gool, W. 2009. "Prevention of Dementia by Intensive Vascular Care (PreDIVA): A Cluster-randomized Trial in Progress," *Alzheimer Disease & Associated Disorders* (23:3), pp. 198–204.

Richard, E., Jongstra, S., Soininen, H., Brayne, C., Moll vav Charante, E. P., and Meiller, Y. 2016. "Healthy Ageing Through Internet Counselling in the Elderly – the HATICE randomised controlled trial for the prevention of cardiovascular disease and cognitive impairment," *BMJ Open*.

Rubin, D. B. 1976. "Inference and Missing Data," *Biometrika* (63:3), pp. 581–592.

Walton, M. K. 2009. "Addressing and Advancing the Problem of Missing Data," *Journal of Biopharmaceutical Statistics* (19:6), pp. 945–956.

Wei, L. 2011. "An Alternative Way to Classify Missing Data Mechanism in Clinical Trials—A Dialogue on Missing Data," *Journal of Biopharmaceutical Statistics* (21:2), pp. 355–361.

Westfall, R. D. 2012. "An Employment-Oriented Definition of the Information Systems Field: An Educator's View," *Journal of Information Systems Education* (23:1), pp. 63–70.

Wittes, J. 2009. "Missing Inaction: Preventing Missing Outcome Data in Randomized Clinical Trials," *Journal of Biopharmaceutical Statistics* (19:6), pp. 957–968.

View publication stats
